# Optical suppression of drug-evoked phasic dopamine release

**DOI:** 10.3389/fncir.2014.00114

**Published:** 2014-09-17

**Authors:** James E. McCutcheon, Jackson J. Cone, Christopher G. Sinon, Samantha M. Fortin, Pranish A. Kantak, Ilana B. Witten, Karl Deisseroth, Garret D. Stuber, Mitchell F. Roitman

**Affiliations:** ^1^Department of Psychology, University of Illinois at ChicagoChicago, IL, USA; ^2^Department of Psychiatry and Department of Cell Biology and Physiology, University of North CarolinaChapel Hill, NC, USA; ^3^Princeton Neuroscience Institute and Department of Psychology, Princeton UniversityPrinceton, NJ, USA; ^4^Departments of Bioengineering, Psychiatry and Behavioral Sciences, Howard Hughes Medical Institute, and CNC Program, Stanford UniversityStanford, CA, USA

**Keywords:** fast-scan cyclic voltammetry, nucleus accumbens, optogenetics, dopamine transients, TH-Cre, rat

## Abstract

Brief fluctuations in dopamine concentration (dopamine transients) play a key role in behavior towards rewards, including drugs of abuse. Drug-evoked dopamine transients may result from actions at both dopamine cell bodies and dopamine terminals. Inhibitory opsins can be targeted to dopamine neurons permitting their firing activity to be suppressed. However, as dopamine transients can become uncoupled from firing, it is unknown whether optogenetic hyperpolarization at the level of the soma is able to suppress dopamine transients. Here, we used *in vivo* fast-scan cyclic voltammetry to record transients evoked by cocaine and raclopride in nucleus accumbens (NAc) of urethane-anesthetized rats. We targeted halorhodopsin (NpHR) specifically to dopamine cells by injecting Cre-inducible virus into ventral tegmental area (VTA) of transgenic rats that expressed Cre recombinase under control of the tyrosine hydroxylase promoter (TH-Cre^+^ rats). Consistent with previous work, co-administration of cocaine and raclopride led to the generation of dopamine transients in NAc shell. Illumination of VTA with laser strongly suppressed the frequency of transients in NpHR-expressing rats, but not in control rats. Laser did not have any effect on amplitude of transients. Thus, optogenetics can effectively reduce the occurrence of drug-evoked transients and is therefore a suitable approach for studying the functional role of such transients in drug-associated behavior.

## Introduction

Generation of burst firing in dopamine neurons is a common feature of all abused drugs (Gysling and Wang, 1983; Mereu et al., 1987; Gessa et al., 1998; Brodie et al., 1999; Koulchitsky et al., 2012). Burst firing results in phasic increases in dopamine in terminal regions, known as dopamine concentration “transients” (Sombers et al., 2009; Owesson-White et al., 2012), which are necessary and sufficient for positive reinforcement and associative learning (Tsai et al., 2009; Steinberg et al., 2013). Transients occur both spontaneously and in response to rewarding or salient environmental stimuli (Day et al., 2007). In addition, they are dramatically enhanced in frequency and amplitude by many abused drugs including ethanol, cocaine, nicotine, and cannabinoids (Cheer et al., 2004, 2007; Aragona et al., 2008; Robinson et al., 2009). Indeed, generation of *de novo* dopamine transients, rather than simply augmenting ongoing ones, seems to be a key feature of all abused drugs (Covey et al., 2014). Thus burst firing and the dopamine concentration transients it produces are likely to play a key role in the development and maintenance of drug-seeking behaviors.

Optogenetics provides a method for determining the role of dopamine transients in positive reinforcement, associative learning and goal directed behaviors including behaviors directed at drugs of abuse. Indeed, mice prefer locations that have been previously paired with phasic, selective activation of dopamine neurons (Tsai et al., 2009). Moreover, rats will self-administer laser pulses that depolarize dopamine cell bodies or dopamine terminals and thus evoke phasic increases in nucleus accumbens (NAc) dopamine (Witten et al., 2011; Steinberg et al., 2014). In stark contrast, locations paired with selective inhibition of dopamine neurons by activation of inhibitory opsins are avoided (Tan et al., 2012; Tye et al., 2013; Danjo et al., 2014). Recently, inhibitory opsins have been used to suppress NAc dopamine release evoked by electrical stimulation of dopamine cell bodies as well as basal levels of NAc dopamine (Danjo et al., 2014). However, the degree to which cell body inhibition via optogenetics affects drug-induced dopamine signaling remains unknown. This is a critical gap in knowledge especially in light of drug effects directly on dopamine terminals (Lüscher and Ungless, 2006) as well as mechanisms at dopamine terminals that can drive release in a cell body independent manner (Exley and Cragg, 2008; Cachope et al., 2012; Threlfell et al., 2012).

Drug-evoked dopamine transients may drive drug-seeking behavior in several ways and transients are known to correlate with operant behaviors to obtain drug (Phillips et al., 2003; Willuhn et al., 2012). However, to date, no studies have assessed the ability of optogenetics to suppress drug-induced, dopamine concentration transients. Here, we use fast-scan cyclic voltammetry to record drug-evoked dopamine concentration transients in NAc while we hyperpolarized the soma of dopamine neurons expressing the inhibitory opsin, halorhodopsin (NpHR). We show that brief somatic hyperpolarization is sufficient to robustly suppress NAc dopamine concentration transients evoked by drugs.

## Materials and methods

### Subjects

Transgenic rats (Long-Evans background) expressing Cre recombinase under the control of the *tyrosine hydroxylase* promoter (TH-Cre^+^ rats; Witten et al., 2011) and wild-type litter mates (TH-Cre^−^ rats) were bred from female TH-Cre^+^ and male wild-type Long-Evans rats (Charles River). All rats used were male and weighed 275–350 g at time of initial surgery. Rats were group-housed until surgery and singly housed thereafter. Standard housing conditions were provided (temperature, 22°C; humidity, 30%; 12:12 h light:dark cycle, lights on at 07:00 h) with *ad libitum* access to food and water. Animal care and use was in accordance with the National Institutes for Health Guide for the Care and Use of Laboratory Animals, and approved by the Institutional Animal Care and Use Committee at the University of Illinois at Chicago.

### Viral infection

The following Cre-dependent viruses (titer, 1.5–4 × 10^12^ particles/mL; serotype AAV5) were purchased from University of North Carolina Vector Core: AAV-EF1a-DIO-eNpHR3.0-EYFP (NpHR); AAV-EF1a-DIO-EYFP (eYFP). TH-Cre^+^ rats infused with NpHR (*NpHR-expressing rats*; *n* = 8) were compared to TH-Cre^+^ rats infused with eYFP or TH-Cre^−^ rats infused with NpHR (*control rats*; *n* = 5).

Rats were anesthetized with ketamine hydrochloride (100 mg/kg, i.p.) and xylazine hydrochloride (10 mg/kg, i.p.), and 2 μL of appropriate virus (see above) was infused using a custom made injector with tips separated 0.8 mm AP and 1 mm DV (adapted from Witten et al., 2011); this permitted two simultaneous infusions at different depths to be made. Coordinates to target the ventral tegmental area (VTA) were as follows (in mm): −5.4 and −6.2 AP, +0.7 ML from Bregma and −8.4 and −7.4 DV from skull surface. 1 μL of virus was infused at each location at a rate of 0.1 μL/min. The infuser was left in place for an additional 8–10 min before being removed. All infusions were made unilaterally. Rats were left in home cage for 6–10 weeks before voltammetry experiments took place.

### Fast-scan cyclic voltammetry and optogenetic suppression

Procedures for recording dopamine release using voltammetry were similar as described previously (Cone et al., 2013). Rats were anesthetized with urethane (1.5–2.0 g/kg, i.p.) and mounted in a stereotaxic instrument. A guide cannula was directed towards the NAc shell (mm from Bregma: +1.7 AP; +0.9 ML) ipsilateral to the viral infusion and a Ag/AgCl reference electrode was placed in contralateral cortex; these were fixed to the skull using screws and dental cement. An optical fiber (Thor Labs; 200 μm, 0.37 numerical aperture, Sparta et al., 2012) was positioned dorsal to VTA (in mm: −5.8 from Bregma; −7.5 to −8.0 from skull surface). A glass-insulated carbon-fiber electrode was lowered into NAc shell using a custom made micromanipulator (UIC Biologic Resources Center). Dopamine concentration transients were evoked by systemic administration (i.p.) of cocaine (10 mg/kg) and raclopride (1 mg/kg), which resulted in production of dopamine transients as previously shown (Venton and Wightman, 2007; Aragona et al., 2008; Park et al., 2010). After establishment of dopamine release, a DPSS laser light source (532 nm; 10–15 mW) was used to illuminate the VTA via the optic fiber for discrete 5 s epochs. Laser power was calibrated before and after every experiment using a power meter (PM100USB; Thorlabs). Voltammetry data were continuously collected in serial 15 s files with the laser turned on for 5 s in every other file. For each rat, 10 “laser on” trials were compared with 10 “laser off” trials.

### Drugs

Cocaine hydrochloride (UIC Pharmacy) and S(−)-raclopride (+)-tartrate salt (Sigma-Aldrich) were dissolved in sterile saline at 10 mg/mL and 1 mg/mL, respectively, before injecting.

### Histology

At the end of recording rats were transcardially perfused. Brains were removed and post-fixed in formalin for 24 h followed by 30% sucrose in 0.1 M phosphate buffer (PB). In a subset of rats, to double-label neurons, anti-TH immunohistochemistry was performed as previously described (McCutcheon et al., 2012). Briefly, 40 μm sections were incubated with anti-TH primary antibody (Millipore, ab152; 1:500) for 72 h at 4°C and with goat anti-rabbit AlexaFluor 594 secondary antibody (Invitrogen, A11037, 1:250) for 90 min at room temperature before being cover-slipped. Images were collected on an Olympus FV1000 microscope.

### Data analysis

Dopamine concentration was extracted from current-voltage plots using principal component analysis (PCA), as previously described (Heien et al., 2004; Keithley et al., 2010). Electrodes were calibrated post-experiment in a custom built flow cell (Sinkala et al., 2012). The mean calibration factor was 54.77 nM/nA.

Dopamine concentration transients were defined as brief elevations in dopamine concentration with fast rise time and decay kinetics similar to those seen after stimulating dopamine cell bodies. Mini Analysis v.6 (Synaptosoft) was used to objectively identify and analyze transients from *concentration x time* traces.

The epoch used for analysis began 1 s after the laser was turned on and ended when the laser was turned off. This was compared to an equivalent epoch in trials when the laser was never turned on. Paired *t*-tests were used to compare “laser on” trials to “laser off” trials. MATLAB was used to conduct statistical testing.

## Results

### Expression of Cre-dependent virus in NAc-projecting dopamine neurons

**Figure 1:**
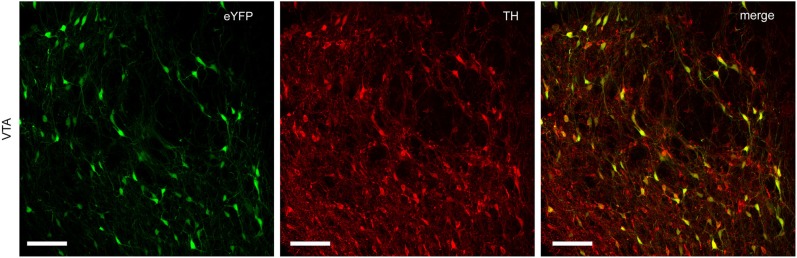
**Specific expression of Cre-dependent transgenes in midbrain dopamine neurons**. TH-Cre^+^ rats infected with Cre-dependent eYFP show a high degree of colocalization between eYFP and TH immunoreactivity in VTA. Left-hand panel, eYFP expression (green); center panel, TH immunoreactivity (red); right-hand panel, merged image (yellow). Scale bar = 100 μm.

**Figure 2:**
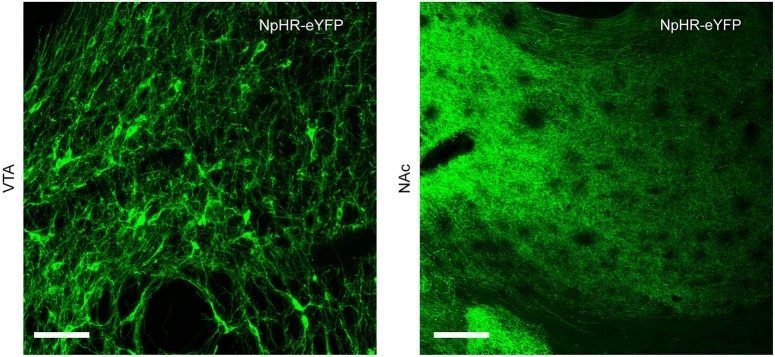
**Neurons expressing Cre-dependent transgenes project to NAc**. TH-Cre^+^ rats infected with Cre-dependent NpHR show expression of eYFP reporter in cell body regions (VTA, left-hand panel) and terminal regions (NAc, right-hand panel). Scale bar = 100 μm.

Infection of TH-Cre^+^ rats with Cre-dependent virus led to expression of transgenes in midbrain dopamine neurons. Expression of eYFP reporter was seen throughout the VTA and substantia nigra compacta of TH-Cre^+^ rats (Figures 1, 2), but not in TH-Cre^−^ rats (data not shown), as expected. Colocalization of eYFP with TH immunoreactivity was observed in the VTA of TH-Cre^+^ rats infected with Cre-dependent eYFP (Figure 1) demonstrating specificity of the transgenic strategy. eYFP intensity was greatest ipsilateral to the viral infusion although, in most animals, some expression was also observed contralaterally. Examination of terminal regions showed that transgene expression was present in neurons projecting to NAc, the site in which voltammetry recordings were made (Figure 2).

### Cocaine and raclopride administration evokes dopamine concentration transients in NAc

Consistent with previous findings (Venton and Wightman, 2007), before drug administration, spontaneous dopamine transients were rarely observed; pre-drug transients were detected in 1 out of 13 rats and in this single rat transient frequency was 1.67 transients/min and mean amplitude was 12.96 ± 0.40 nM. Co-administration of cocaine and raclopride led to generation of spontaneous, high amplitude transients in 11 out of 13 rats. In these 11 rats, mean transient frequency after drug was 10.31 ± 1.63 transients/min and mean amplitude was 31.93 ± 3.91 nM. Transients began approximately 10 min after drug administration and continued at a similar rate until the end of the experiment, at least 2 h.

### Optical suppression of drug-evoked dopamine release

**Figure 3:**
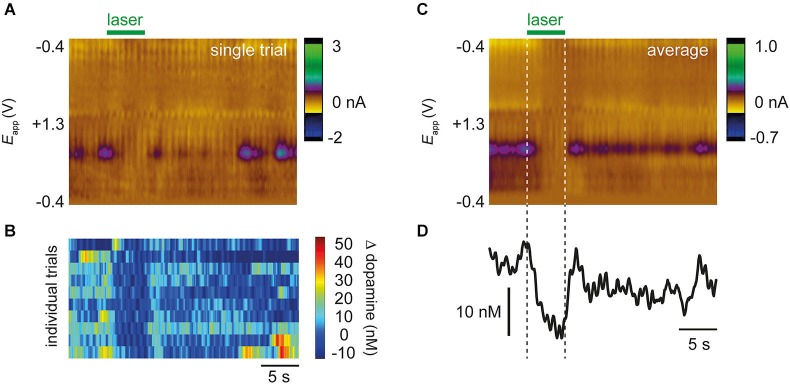
**Representative data from a single rat showing optical suppression of drug-evoked dopamine transients**. **(A)** Color plot from single trial after administration of cocaine and raclopride. Applied voltage is on *y*-axis, time is on *x*-axis, and current shown as pseudocolor. Purple features correspond to dopamine oxidation (~+0.6 V). Laser decreases the occurrence of dopamine transients. **(B)** 10 individual trials from the same rat showing that the laser consistently reduces occurrence of transients. **(C)** Average color plot produced from 10 trials in **(B)**. Conventions are the same as in **(A)**. **(D)** Dopamine concentration extracted from color plot using principal component analysis.

**Figure 4:**
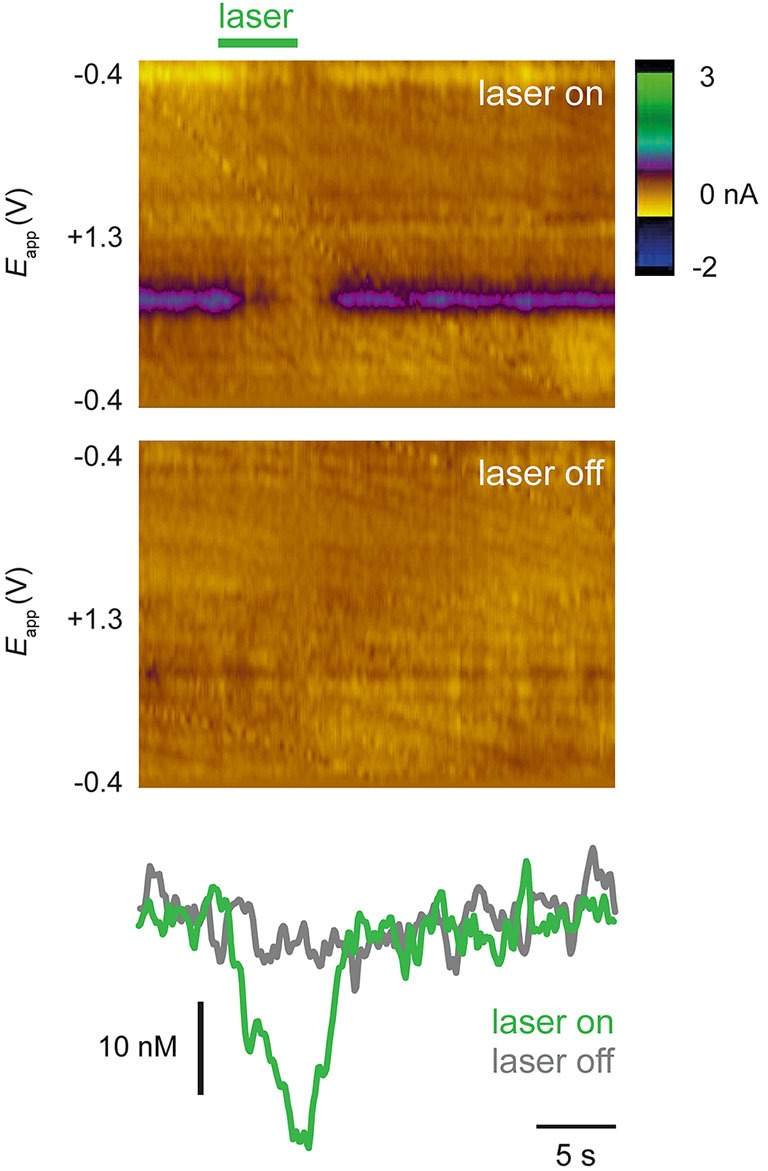
**A steady-state pattern of dopamine release is unmasked by optical suppression**. Recordings from a rat in which no transients or dopamine release were observed until laser was administered. Top color plot (conventions as in Figure 3A) shows a single trial in which laser was switched on (green bar) and bottom color plot shows single trial in which laser was not switched on. Bottom panels show dopamine concentration traces extracted from color plots. In this rat, steady state dopamine release was not evident until laser was used to activate NpHR.

Approximately 30 min after drug administration, an optic fiber was used to illuminate the VTA and activate NpHR expressed in dopamine cells. In NpHR-expressing rats, the laser strongly suppressed dopamine release. Figure 3 shows dopamine transient analyses in a representative NpHR-expressing rat. Reduction of dopamine transients was seen robustly from trial to trial (Figure 3B). Interestingly, in an NpHR-expressing rat in which no transients were observed, the laser was still able to reduce dopamine concentration, and revealed the presence of a steady-state level of dopamine, which would not have been detected otherwise (Figure 4).

**Figure 5:**
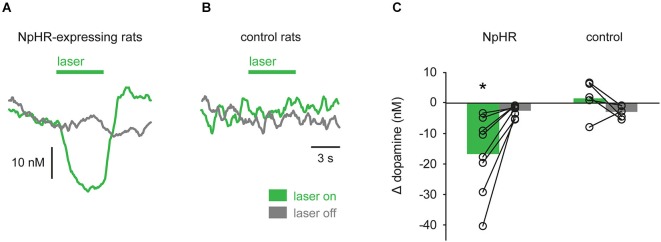
**Group data showing optical suppression of dopamine signaling**. **(A)** In TH-Cre^+^ rats transfected with NpHR (*n* = 8), laser illumination of VTA strongly suppressed dopamine, relative to trials in which the laser was not turned on. **(B)** No effect of laser was seen in control rats (*n* = 5). **(C)** Quantification of data in **(A)** and **(B)**. Circles are individual rats and bars are mean values. * *p* < 0.05 laser on vs. laser off.

To analyze the effect of the laser, mean dopamine concentration during the laser epoch was subtracted from mean dopamine concentration during a corresponding baseline epoch that preceded the laser (“laser on” trials; see Section Materials and Methods). This value was compared with a similar calculation performed on trials in which the laser was not turned on (“laser off” trials). Analysis of all NpHR-expressing rats shows a greater suppression of dopamine concentration during “laser on” trials, compared with “laser off” trials (Figure 5A; *t*_(7)_ = −3.489, *p* = 0.010). Control rats were defined as TH-Cre+ rats injected with eYFP reporter or TH-Cre^−^ rats injected with NpHR. No difference between “laser on” and “laser off” trials was observed in these rats and they were pooled for analysis (Figure 5B; *t*_(4)_ = 1.569, *p* = 0.192).

**Figure 6:**
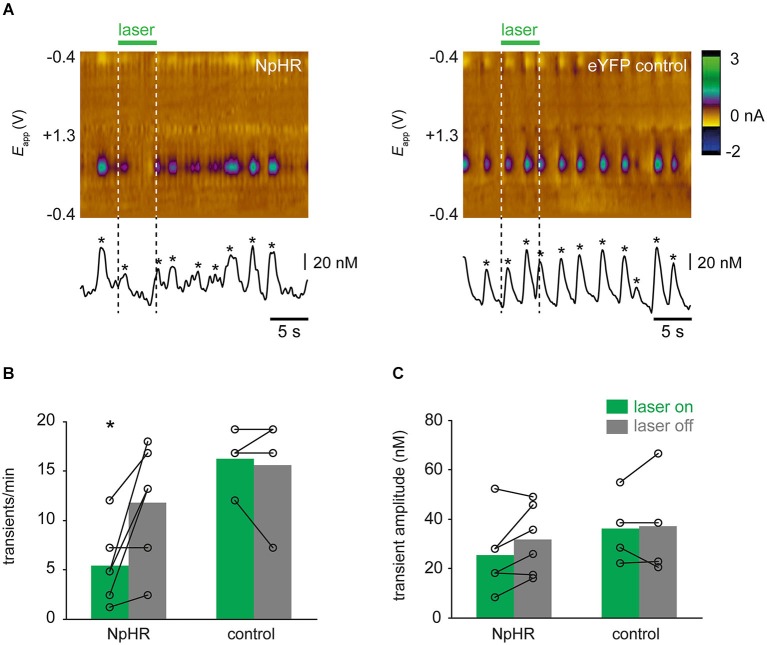
**Optical suppression of dopamine transients**. **(A)** Single trial examples showing effect of laser on dopamine transients. Color plots and concentration traces (conventions as in Figure 3A) are shown from an NpHR-expressing rat (left-hand panel) and an eYFP-expressing control rat (right-hand panel). Asterices show detected transients. **(B)** Frequency of dopamine transients is reduced during laser illumination of VTA, relative to trials in which laser was not turned on, in TH-Cre^+^ rats expressing NpHR (*n* = 7), but not control rats (*n* = 4). * *p* < 0.05 laser on vs. laser off. **(B)** Amplitude of transients was not significantly affected by laser illumination in either group.

In rats in which dopamine transients were observed, characteristics of transients were further analyzed. One control rat exhibited very low spontaneous activity (<1 transient/min) and was excluded from this analysis. In NpHR-expressing rats, the frequency of transients was reduced during “laser on” trials, compared with corresponding “laser off” trials (Figure 6A; *t*_(5)_ = −2.961, *p* = 0.031). This reduction in frequency was not seen in control rats (*t*_(3)_ = 0.397, *p* = 0.718). Surprisingly, even though transient frequency was reduced during “laser on” trials in NpHR-expressing rats, when transients were detected, their amplitude was not affected by the laser (Figure 6B; *t*_(5)_ = −2.014, *p* = 0.100). No change in transient amplitude was seen in control rats as a result of laser (*t*_(3)_ = −0.259, *p* = 0.813).

## Discussion

Here, we show that somatic hyperpolarization using optogenetics is sufficient to suppress drug-evoked phasic dopamine transients. Specifically, by illuminating dopamine neurons that were selectively transfected with the inhibitory opsin NpHR, we robustly attenuated the occurrence of dopamine transients in NAc produced by co-administration of cocaine and raclopride. We used this pharmacological challenge as it is known to potently drive phasic dopamine release (Venton and Wightman, 2007; Aragona et al., 2008; Park et al., 2010). Fast-scan cyclic voltammetry was used to measure dopamine *in vivo* due to its sub-second time resolution, which permits detection of brief release events. This study paves the way for future studies using optogenetics to discern the role of dopamine transients in driving drug reinforcement and drug-seeking behaviors.

In this study we recorded from NAc rostral shell. We chose this region as it exhibits a high degree of spontaneous and drug-evoked transient activity (Aragona et al., 2008; Roitman et al., 2008; Park et al., 2010; Daberkow et al., 2013). In addition, previous experiments with transgenic TH-Cre^+^ rats used virus infusion coordinates that targeted medial VTA (Witten et al., 2011; Steinberg et al., 2013); of the striatal subregions, medial VTA sends densest projections to NAc shell (Ikemoto, 2007). Correspondingly, we found that eYFP expression in terminals was densest, in most rats, in NAc shell. It is unlikely that the efficacy of light to hyperpolarize dopamine neurons and suppress dopamine transients would be different in neurons with different projection targets. Future experiments will confirm if this is indeed the case.

We observed suppression of dopamine release in all NpHR-expressing rats. This finding is consistent with cocaine and raclopride stimulating burst firing in dopamine neurons (Shi et al., 2004) and somatic inhibition suppressing that activity. However, in most cases, we did not block dopamine signaling altogether. That is, while the frequency of transients was reduced during laser epochs transient production was not completely abolished. Indeed, when transients did occur during laser epochs, transient amplitude was not affected. There may be several reasons for this. First, expression of the virus in all dopamine cells was likely not complete due to penetrance of the transgenic strategy, efficiency of the virus, and slight variance in infusion site and virus diffusion. As such, it is possible that the greater degree of suppression seen in some animals was due to a greater opsin expression; in other studies using TH-Cre^+^ rats, opsin expression has been shown to correlate with behavioral outcome (Witten et al., 2011; Steinberg et al., 2014). Second, the recruitment of infected neurons by light may not have been complete. The VTA is a relatively large area in the rat and light scattering could be problematic. Indeed the degree of light scattering differs depending on brain region (Al-Juboori et al., 2013). Third, it is worth noting that the stimulus we used to evoke dopamine release—co-administration of cocaine and raclopride—is likely to have activated the dopamine system in a supra-physiological manner and this may have prevented optical inhibition of all dopamine release. Indeed, as in this paper we did not directly measure effects on firing we cannot confirm that activation of the NpHR-mediated chloride conductance will completely block spike generation; rather, it may have simply decreased the probability of firing. Finally, and perhaps most intriguing, terminal mechanisms for dopamine release may remain intact even during somatic optical inhibition and act independently of activity in the soma (Exley and Cragg, 2008; Cachope et al., 2012; Threlfell et al., 2012). For example, cocaine increases the activity of NAc cholinergic interneurons (Witten et al., 2010). NAc acetylcholine, in turn, is capable of inducing dopamine release at terminals (Cachope et al., 2012; Threlfell et al., 2012). This last point is especially intriguing in that somatic optical suppression may be a means to determine the relative contributions on somatic vs. terminal influences on NAc dopamine signaling. Thus, it is conceivable that the frequency of transients correlates with somatic burst firing, whereas amplitude of each transient may be more subjected to terminal regulation by dopamine transporters and autoreceptors. Future studies will follow up on this by comparing somatic and terminal activation of opsins.

We observed an increase in dopamine concentration and the probability of a dopamine transient immediately after termination of the laser in some rats, which could be characterized as an inhibition-induced rebound in dopamine concentration. This rebound response could reflect either a property of NpHR (e.g., change in chloride reversal potential after sustained channel opening; Raimondo et al., 2012) or an intrinsic property of dopamine neurons. Consistent with the latter, the response is reminiscent of the brief increase in firing of dopamine cells seen following the termination of an aversive stimulus in some electrophysiology experiments (Brischoux et al., 2009; Wang and Tsien, 2011). The rebound excitation has been suggested to signal relief at termination of an aversive stimulus and could be used by an organism to motivate behavior to learn about aversive events and avoid future occurrences.

Several studies have assessed the contribution of cell body excitability on dopamine transients monitored in dopamine terminal regions. Sombers et al. (2009) used intra-VTA lidocaine or the NMDA receptor antagonist, 2-amino-5-phosphonopentanoic acid, and found near complete suppression of spontaneous and drug-induced dopamine transients. This was replicated with lidocaine in a more recent report (Owesson-White et al., 2012). In contrast, intra-VTA infusion of GABA receptor agonists baclofen and muscimol had no effect on spontaneous transients but blocked the cocaine-evoked increase in transient frequency (Aragona et al., 2008). Although these methods were generally efficient at blocking transients, the use of optogenetics provides an unprecedented degree of both temporal and neuronal population specificity. Thus, optogenetic suppression of drug-induced transients allows the contribution of dopamine neuron excitability to be isolated while leaving GABAergic and other cell types responsive to drug influences. Moreover, it permits the silencing of drug-induced transients at brief times that are governed by the experimenter.

A number of studies have used optogenetics to suppress firing of dopamine neurons either by directly targeting dopamine neurons with an inhibitory opsin (Tan et al., 2012; Tye et al., 2013; Danjo et al., 2014; Ilango et al., 2014) or by stimulating GABAergic afferents to dopamine neurons using channelrhodopsin (Stamatakis and Stuber, 2012; Tan et al., 2012; van Zessen et al., 2012; Jennings et al., 2013). In most of these studies, optogenetic suppression of dopamine activity was verified by testing the ability of optical stimulation to suppress action potential firing *in vivo* or evoke hyperpolarizing currents in dopamine neurons in an *ex vivo* slice preparation (Tan et al., 2012; van Zessen et al., 2012; Chaudhury et al., 2013; Jennings et al., 2013; Danjo et al., 2014). Two of these studies used fast-scan cyclic voltammetry to examine dopamine release in terminal regions: van Zessen et al. (2012) showed a reduction in electrically-evoked dopamine release caused by activation of GABAergic inputs to dopamine cells and Danjo et al. (2014) showed a reduction in electrically-evoked dopamine release and tonic levels of dopamine caused by activation of the inhibitory opsin, ArchT, in dopamine neurons. Our work significantly extends these findings to optical suppression of drug-induced dopamine concentration transients. While our work was conducted in anesthetized rats, the drug cocktail (cocaine and raclopride) delivered has been shown to robustly stimulate dopamine concentration transients in both anesthetized (Park et al., 2010) and awake, behaving rats (Aragona et al., 2008). Future work will address whether optical suppression of drug-evoked dopamine concentration transients is sufficient to prevent the development of drug-directed behavior. Interestingly, a different approach to dopamine neuron activity modulation using a designer receptor exclusively activated by designer drug (DREADD) has been shown to affect reinstatement for drug seeking (Mahler et al., 2014).

Optogenetic inhibition of dopamine cells increased immobility in the tail suspension test and reduced sucrose preference (Tye et al., 2013), or induced a real-time and conditioned place avoidance (Tan et al., 2012; Danjo et al., 2014; Ilango et al., 2014). Indirect suppression of dopamine neurons via GABAergic afferents disrupts reward consumption (van Zessen et al., 2012), induces real-time place avoidance, and marks the onset of anxiety-like behavior in an open field (Stamatakis and Stuber, 2012; Jennings et al., 2013). Thus, optogenetic disruption of dopamine cell activity during behavior results in robust behavioral outcomes, many of which resemble an aversive state (although see Chaudhury et al., 2013). That optical suppression of dopamine release induces indices of depression and aversion matches well with real-time recordings made of NAc dopamine concentration in response to aversive stimuli (Roitman et al., 2008; Wheeler et al., 2011; McCutcheon et al., 2012).

In summary, somatic hyperpolarization of dopamine neurons is sufficient to suppress the occurrence of dopamine transients in terminal regions. As *de novo* generation of dopamine transients is a key feature common to all abused drugs (Covey et al., 2014), that optical somatic inhibition can suppress drug-induced dopamine transients offers new opportunities to control drug reinforcement and drug-seeking behaviors. Future studies will use optogenetics to determine the precise contribution of drug-evoked transients to drug-associated behaviors.

## Conflict of interest statement

The authors declare that the research was conducted in the absence of any commercial or financial relationships that could be construed as a potential conflict of interest.
